# An Electronic Medical Record–Based Prognostic Model for Inpatient Falls: Development and Internal-External Cross-Validation

**DOI:** 10.2196/59634

**Published:** 2024-11-13

**Authors:** Rex Parsons, Robin Blythe, Susanna Cramb, Ahmad Abdel-Hafez, Steven McPhail

**Affiliations:** 1 Australian Centre for Health Services Innovation and Centre for Healthcare Transformation School of Public Health and Social Work Queensland University of Technology Kelvin Grove Australia; 2 Jamieson Trauma Institute Royal Brisbane and Women's Hospital Metro North Health Herston Australia; 3 College of Computing and Information Technology University of Doha for Science and Technology Doha Qatar; 4 Clinical Informatics Directorate Metro South Health Woolloongabba Australia

**Keywords:** clinical prediction model, falls, patient safety, prognostic, electronic medical record, EMR, intervention, hospital, risk assessment, clinical decision, support system, in-hospital fall, survival model, inpatient falls

## Abstract

**Background:**

Effective fall prevention interventions in hospitals require appropriate allocation of resources early in admission. To address this, fall risk prediction tools and models have been developed with the aim to provide fall prevention strategies to patients at high risk. However, fall risk assessment tools have typically been inaccurate for prediction, ineffective in prevention, and time-consuming to complete. Accurate, dynamic, individualized estimates of fall risk for admitted patients using routinely recorded data may assist in prioritizing fall prevention efforts.

**Objective:**

The objective of this study was to develop and validate an accurate and dynamic prognostic model for inpatient falls among a cohort of patients using routinely recorded electronic medical record data.

**Methods:**

We used routinely recorded data from 5 Australian hospitals to develop and internally-externally validate a prediction model for inpatient falls using a Cox proportional hazards model with time-varying covariates. The study cohort included patients admitted during 2018-2021 to any ward, with no age restriction. Predictors used in the model included admission-related administrative data, length of stay, and number of previous falls during the admission (updated every 12 hours up to 14 days after admission). Model calibration was assessed using Poisson regression and discrimination using the area under the time-dependent receiver operating characteristic curve.

**Results:**

There were 1,107,556 inpatient admissions, 6004 falls, and 5341 unique fallers. The area under the time-dependent receiver operating characteristic curve was 0.899 (95% CI 0.88-0.91) at 24 hours after admission and declined throughout admission (eg, 0.765, 95% CI 0.75-0.78 on the seventh day after admission). Site-dependent overestimation and underestimation of risk was observed on the calibration plots.

**Conclusions:**

Using a large dataset from multiple hospitals and robust methods to model development and validation, we developed a prognostic model for inpatient falls. It had high discrimination, suggesting the model has the potential for operationalization in clinical decision support for prioritizing inpatients for fall prevention. Performance was site dependent, and model recalibration may lead to improved performance.

## Introduction

Falls in hospitals cause serious injuries and deaths [1]. Inpatient falls are difficult to predict [2] or prevent [3], although some inpatient fall prevention strategies that require efforts early in patients’ admissions have been effective [4-6]. The use of health professional–completed fall risk assessment and screening tools to identify patients at high risk of falls has been a popular approach to stratify the risk of falling for inpatients, as has the development of clinical prediction models incorporating these assessments [7,8]. A clinical prediction model is a model that estimates an individual’s probability of a current health condition (diagnostic) or one that may occur in the future (prognostic). These models are usually estimating a probability, or risk, for the given patient and health outcome. As a result, a prognostic model is a form of a clinical prediction model (or risk prediction model) that would be relevant to estimating the risk of falling for an inpatient in a manner that could guide the use of a fall prevention strategy by clinical teams. Recent efforts include work to use prediction tools to guide the selection of specific prevention strategies to prevent inpatient falls [9]. However, the discriminatory performance of inpatient falls risk assessment tools and prediction models is often poor, which may have contributed to why many risk model–guided treatments have failed to demonstrate effectiveness [9]. This study used the Northern Hospital Modified St Thomas’ Risk Assessment Tool, which was developed for acute inpatients and had a sensitivity and specificity of 0.65 and 0.79, respectively [10]. A recent multihospital trial demonstrated the noninferiority and potential superiority of divestment away from conventional in-hospital fall risk assessment toward a simple clinical decision support that prompted consideration of potential patient interventions [11]. The tools used in both of these studies required clinical teams to complete questionnaires and manually score patient risk based on tallying up the identified risk factors. Conventional fall risk assessments may therefore be introducing additional data collection burdens to health professionals without leading to better patient outcomes.

Our previous review that investigated the methods and data used to develop prognostic models for inpatient falls found that reporting quality was typically poor, with a largely homogeneous approach to model development [8]. No studies applied time-to-event methods, also known as survival modeling approaches, to predict falls. Instead, studies typically used data available at admission to predict a binary end point of fall at any time during the patient admission over unknown and heterogenous lengths of time [8]. This approach has the potential to be misleading if used in a clinical decision support system, as the estimated risk (probability of a fall) may be primarily driven by exposure time through long lengths of stay rather than the proximal risk of experiencing a fall event. If exposure time is not appropriately accounted for during model development, fall prevention strategies may be disproportionately assigned to those who are likely to stay longer at the expense of those who are at an elevated relative risk despite shorter anticipated lengths of stay.

Many existing fall prediction models rely upon a wide variety of data sources, often requiring manual collection and data entry, which limits the potential for integration into real-time risk prediction and clinical decision support [12]. Development of a robust, interpretable prediction model that can estimate the risk of inpatient falls using solely routinely recorded electronic medical record (EMR) data to guide the assignment of effective inpatient fall prevention strategies remains a priority. An important anticipated advantage of relying on routinely recorded EMR data is that risk estimates can be generated without the requirement for additional fall risk screening tasks to be completed by clinical staff, likely being resource-saving when compared with widely used questionnaire-based falls risk screening. It is also particularly important for the first days of an admission period when in-hospital fall prevention strategies are typically initiated and when most falls occur, as risk estimates can be generated immediately as data are available [13].

Primarily, our previous review highlighted that relying on data collected as part of routine clinical care and stored within EMR systems and using time-to-event modeling approaches is underexplored and may be particularly useful and relevant for the given clinical context of falls risk screening, assessment, and prevention [8]. Our primary interest was to develop a prognostic model for inpatient falls using a survival modeling approach and EMR data from 5 hospitals that performed well early in admission, with the potential for implementation in a clinical decision support tool to help augment clinician decision-making regarding the initiation of effective inpatient fall prevention interventions.

This study is reported in accordance with the Transparent Reporting of a Multivariable Prediction Model for Individual Prognosis or Diagnosis (TRIPOD) statement and checklist [14].

## Methods

### Overview

Model development choices were informed by a previous review [8] and best-practice recommendations [15,16]. The model was developed using Cox proportional hazards regression and included time-varying covariates and predictor variables available within the EMR system. Performance was assessed in terms of discrimination and calibration using internal-external cross-validation, and the final model was fit using the full dataset.

### Setting and Data Cleaning

After consultation with fall prevention experts and hospital geriatricians, we censored observations after 14 days of admission. After 14 days, hospital falls occurred at a lower rate and may be associated with a different clinical context for long-stay patients that may benefit from a more targeted modeling strategy. To ensure the data focused on the period in which fall interventions are initiated and most falls occur, we used EMR data for the first 14 days of admission from patients admitted to 5 Australian hospitals between 2018 and 2021 to develop our model. Our intention was for study findings to be representative of inpatient hospital wards broadly. There were no exclusion criteria applied to eliminate particular subgroups of hospital patients; patients from any ward and of any age were included for model development and validation. In each of our included hospitals, inpatient falls were recorded in the EMR using RiskMan (RiskMan International Pty Ltd), a proprietary patient safety system for tracking and managing adverse events [17]. RiskMan reports were linked to each inpatient admission record using a unique identifier, including date and time stamps. When the time of fall was not recorded, it was assumed to be at midnight on the morning of the date specified to ensure no predictions used data recorded after the fall.

To maximize the potential for generalizability and ease of potential transferability to external hospital systems while eliminating the need for clinical staff time for additional fall risk assessment and data entry, predictor variables used in the model were primarily derived from administrative information routinely recorded for all hospital admissions. This included the source of the admission (eg, whether they came from residential aged care or through the emergency department), type of admitting medical service (eg, neurosurgery or orthopedics), sex (coded as the binary variable “Female”), age in years, as well as years since 2018 to incorporate potential adjustment for longer-term trends in fall risk over the study period. We also included 2 time-varying covariates in the model—the number of previous inpatient falls during the current admission up until the time of prediction and the time since admission, in hours. The time since admission variable was updated every 12 hours throughout the admission.

Because coding for admission source and medical service categories with similar or overlapping meanings varied across hospital facilities, we aggregated these into overarching categories that could be readily applied to data from all hospitals based on clinical groupings, shown in Table 1. Using these overarching clinical groupings also mitigated the risk of categorical variables for admission source or medical service being exclusive to the training or validation datasets. The “Other” categories for these 2 variables were used to include missing data as well. There were no missing data for other covariates included in the model. Restricted cubic splines with 4 knots were used for patient age and the years since 2018, and a restricted cubic spline with 3 knots was used for the hours since admission variable. A spline function with *n* knots is made up of *n*+1 piecewise polynomial functions where the knot locations describe the place (value of the variable to which spline is being applied) where these 2 sequential polynomial functions meet. For example, in the full model, the restricted cubic spline applied to patient age has 4 knots, meaning that there are 5 polynomial functions that meet at the 4 locations (values of patient age; described in Multimedia Appendix 1). Further information on use and interpretation of spline terms are illustrated in Regression Modeling Strategies by Harrell [18]. The restricted cubic splines used in this study have an advantage for use in prediction models in that they are constrained to be linear in their 2 tails (above the last knot and before the first knot). This is particularly important considering the use of “years since 2018” as a predictor and the possible use of the model in years that exceed the range of the data used to fit it.

**Table 1:** Demographics and summary of key predictor variables used in the model.

Variable and measure			Hospital										All
			1		2	3		4		5			
**Inpatient admissions, n**													
	Total	462,891		359,075		150,458	124,149		10,983		1,107,556		
	Days	1,198,736		612,658		256,887	273,179		31,882		2,373,343		
	Days (truncated at 14 days)	794,398		485,189		197,951	228,770		23,825		1,730,133		
**Female, n (%)^a^**			191,732 (41.42)		205,809 (57.32)	89,113 (59.23)		65,440 (53.71)		7364 (67.05)		559,458 (50.51)	
**Falls (truncated at 14 days)**													
	Total, n	3027		1377		638	828		134		6004		
	Fallers, n (%)^a^	2678 (0.58)		1248 (0.35)		562 (0.37)	731 (0.59)		122 (1.11)		5341 (0.48)		
**Age (year), median (IQR)**			63 (48-74)		42 (26-63)	52 (31-72)		61 (43-77)		38 (26-71)		56 (35-72)	
**Length of stay (days), median (IQR)**			0.3 (0.1-1.3)		0.3 (0.2-1.2)	0.3 (0.2-1.2)		0.4 (0.2-2.1)		1.0 (0.2-2.3)		0.3 (0.2-1.4)	
**Medical service, n**													
	General medicine	22,837		26,694		22,678	22,869		5042		100,120		
	Orthopedics	15,833		10,694		<100^b^	8138		<100		34,665		
	Psychiatry	16,932		12,209		2729	<100		<100		31,870		
	Rehabilitation	7840		905		<100	8912		<100		17,657		
	Neurosurgery	5449		<100		<100	<100		<100		5449		
	Palliative medicine	<100^a^		1553		613	1573		<100		3739		
	Geriatrics	2145		<100		<100	<100		<100		2145		
	Psychogeriatric	628		<100		<100	<100		<100		628		
	Spinal	378		<100		<100	<100		<100		378		
	Other	390,849		307,020		124,438	82,657		5941		910,905		
**Admission source, n**													
	Emergency department—this hospital	128,782		182,224		77,243	84,570		3990		476,809		
	Outpatient department—this hospital	208,239		91,621		38,291	29,378		4160		371,689		
	Routine readmission no referral required	84,698		63,475		25,576	7002		327		181,078		
	Admitted patient transferred from other hospital	20,187		3906		1186	2084		744		28,107		
	Private medical practitioner (not psychiatry)	11,119		136		340	616		<100		12,221		
	Residential aged care service	2140		<100		<100	<100		<100		2166		
	Community service	257		1237		237	363		<100		2130		
	Other	7469		16,471		7582	118		1716		33,356		

^a^Percentages were calculated with the total number of inpatient admissions as the denominator.

^b^Cells with small values have been replaced with “<100” to address privacy concerns.

### Evaluation

Following advice from Steyerberg and Harrell [16], we developed the model using the full dataset but evaluated model performance using internal-external cross-validation. Internal-external cross-validation is a process in which the folds used for cross-validation correspond to individual study sites, in this case, hospitals. This method is useful for reporting prediction performance, as allocation to validation folds is nonrandom, is reproducible, and provides better estimates of model generalizability [19]. It also means that every validation fold is an entirely separate hospital from those used for fitting the model, meaning that it provides a better estimate of generalizable performance to new settings than internal validation (where data from a single site are divided into a development dataset and an evaluation dataset). Table 2 shows a summary of how the internal-external cross-validation folds are constructed from the dataset comprising 5 hospitals. In this process, 5 models are fit—one for each hospital being used as a validation dataset, with the remaining 4 hospitals being used to fit the model. Table 2 shows a summary of the data used to fit each of the 5 models (fold 1 to 5) and the final model (fit using the data from all hospitals). Using the entire dataset for model development but internal-external cross-validation for reporting ensures that the model does not suffer from avoidable losses in performance by using a subset of the data and potentially omitting useful observations [16].

Model performance was evaluated in terms of discrimination and calibration. The area under the time-dependent receiver operating characteristic (ROC) curve was calculated for each fold and a “combined” curve was derived to show the moving average across folds. The time-dependent ROC curve was evaluated using prediction times of every 24 hours from 1 day post admission to 1 week after admission. The *survivalROC* R package [20] was used to estimate the time-dependent ROC curves. To estimate the parameter uncertainty of our performance metrics, we applied the approach described by LeDell and colleagues [21] by modifying the *cvAUC* R package [22] to combine area under the curve (AUC) values from multiple cross-validation folds and construct 95% CIs. For visualization purposes, the curves labeled “combined” on the ROC plots in this study were constructed using vertical averaging [23].

To evaluate model calibration, we followed advice from McLernon and colleagues [24] to obtain moderate calibration curves over the full-time range. As described in the supplement of their study, we used a Poisson model with the outcome regressed against the linear predictors, fit with a restricted cubic spline, and the log of the predicted cumulative hazard as an offset. Smoothed curves were applied to investigate whether observed fall event rates were equal to the predicted risk among patients with the same predicted risk. We created curves separately for each validation fold.

**Table 2:** Sample size calculations and summary.

Model^a^	Inpatient admissions, n	Patient days (truncated at 14 days), n	Falls, n	Model parameters, n	Events per parameter, n
Fold: 1	644,665	935,735	2977	22	135
Fold: 2	748,481	1,244,944	4627	26	178
Fold: 3	957,098	1,532,182	5366	26	206
Fold: 4	983,407	1,501,363	5176	26	199
Fold: 5	1,096,573	1,706,308	5870	26	226
Final	1,107,556	1,730,133	6004	26	231

^a^Model represents the cross-validation fold models and the final model fit with all patient data. The fold models are those that fit during internal-external cross-validation and incorporate all patient data except for the associated hospital of the same number. For example, the “Fold: 1” model was fit using patient data from hospitals 2 to 5, with hospital 1 being the validation set.

### Sample Size

We considered a model with a possible 30 parameters, a fall rate of 0.0054, a conservative estimate of 0.143 Cox-Snell *R*^2^ for sample size calculation, a selected prediction time of 24 hours, and mean follow-up of 37.5 hours (the mean length of stay within the truncated dataset). The Cox-Snell *R*^2^ was obtained by using an estimated C-index of 0.7, the approximate performance of recently published inpatient fall prediction models [8], and transforming it into an estimate for the Cox-Snell *R*^2^ using methods described in related works by Jinks et al [25] and Royston and Sauerbrei [26]. Using the *pmsampsize* R package [27], we estimated that the minimum sample size for fitting this Cox model would be 1619 patients, with 330 fall events, and 11.75 events per parameter. During our internal-external cross-validation, the least powered model that was fit included 2977 falls and 135 events per parameter (22 parameters; a summary of model parameters, patients, and events used for each model fit during cross-validation is illustrated in Multimedia Appendix 2). The final model was fit with 6004 falls and 231 events per parameter (26 parameters).

### Ethical Considerations

Approval was granted by the Metro South Human Research Ethics Committee (HREC/2020/QMS/64807). The requirement for informed consent was waived.

To protect the privacy and confidentiality of candidates, the data used in the study cannot be publicly shared. However, analysis code, including software and package versions, is available on GitHub [28].

## Results

Patient characteristics and frequencies for admission source and medical service are described for each included hospital in Table 1. The admitted patient records over 4 years (2018 to 2021) used in this study included 1,107,556 inpatient admissions. There was approximately even representation of male and female patients (559,458/1,107,556, 50.51% female) and the median (IQR) age was 56 (35-72) years. There were 6004 falls and 5241 individual fallers.

There were expected and notable differences in patient age and sex characteristics between hospitals, consistent with the clinical services offered at each facility and populations served. In this context, hospitals differed by size and level of intensive care unit services, specialized geriatric care units, rural or inner-city location, and number of beds (Table 1) [29].

The final model fit to the entire dataset is provided in Table 3 with associated locations for spline terms in Multimedia Appendix 1. This study was conducted with the primary aim of developing a prognostic model for inpatient falls, not to identify prognostic factors. Interpreting estimates from Table 3 allows us to identify which factors were associated with higher risk estimates but should not be interpreted as causally related to falls. Male patients, patients receiving geriatric or rehabilitation care, and those with previous falls were associated with higher risk estimates.

**Figure 1:**
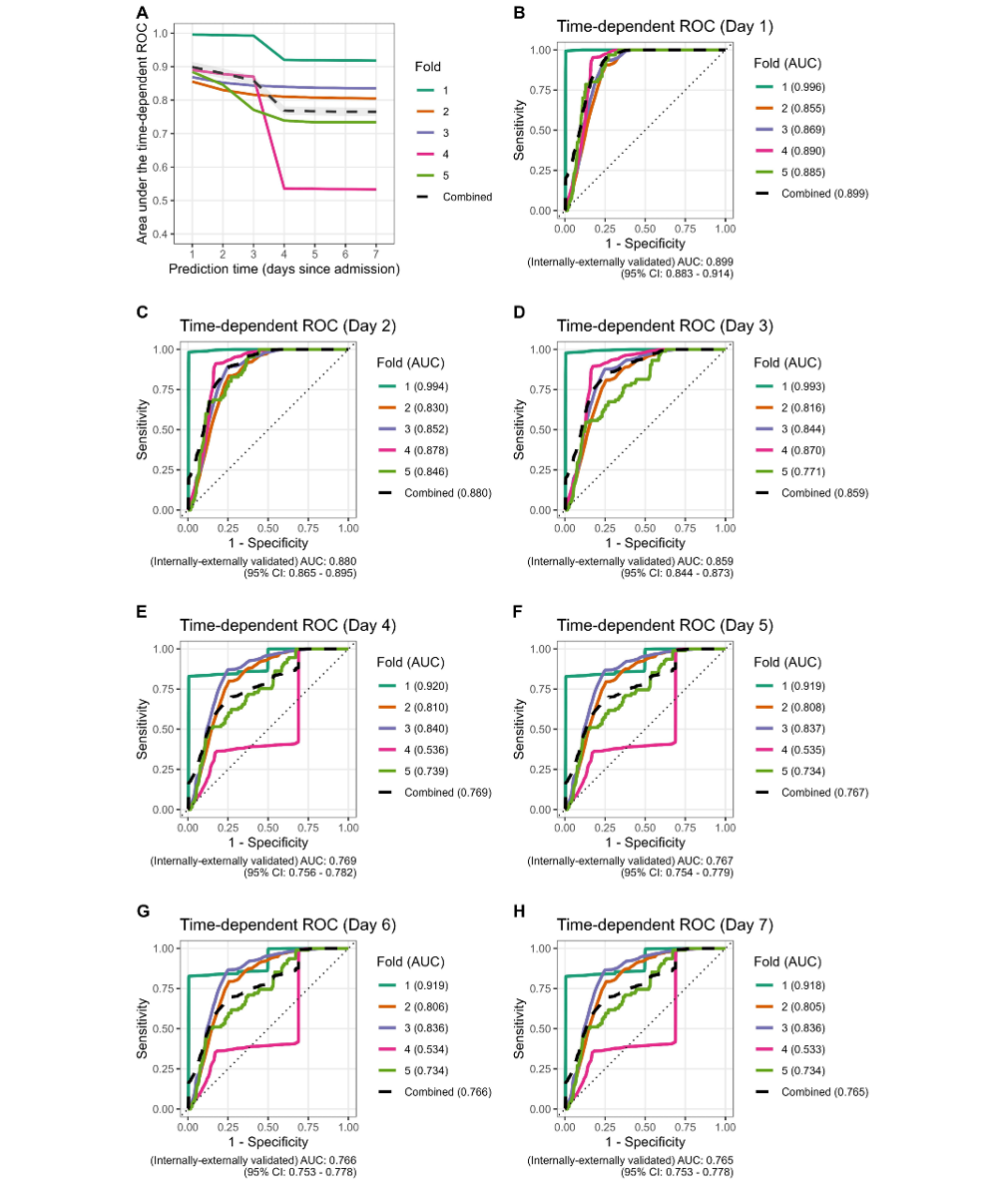
Discrimination performance. (A) Time series of discrimination performance for prediction times between 1 day and 1 week. The colored lines represent performance at difference centers when used as the holdout set during cross-validation, and the dashed black line represents the aggregate measure of discrimination with the gray region as the 95% CI for this estimate. (B-H) Time-dependent ROC curves for predicted times from 1 day to 1 week, each including a curve for each of the 5 models fit during internal-external cross-validation as well as a “combined” curve derived using vertical-averaging. AUC: area under the curve; ROC: receiver operating characteristic.

**Figure 2:**
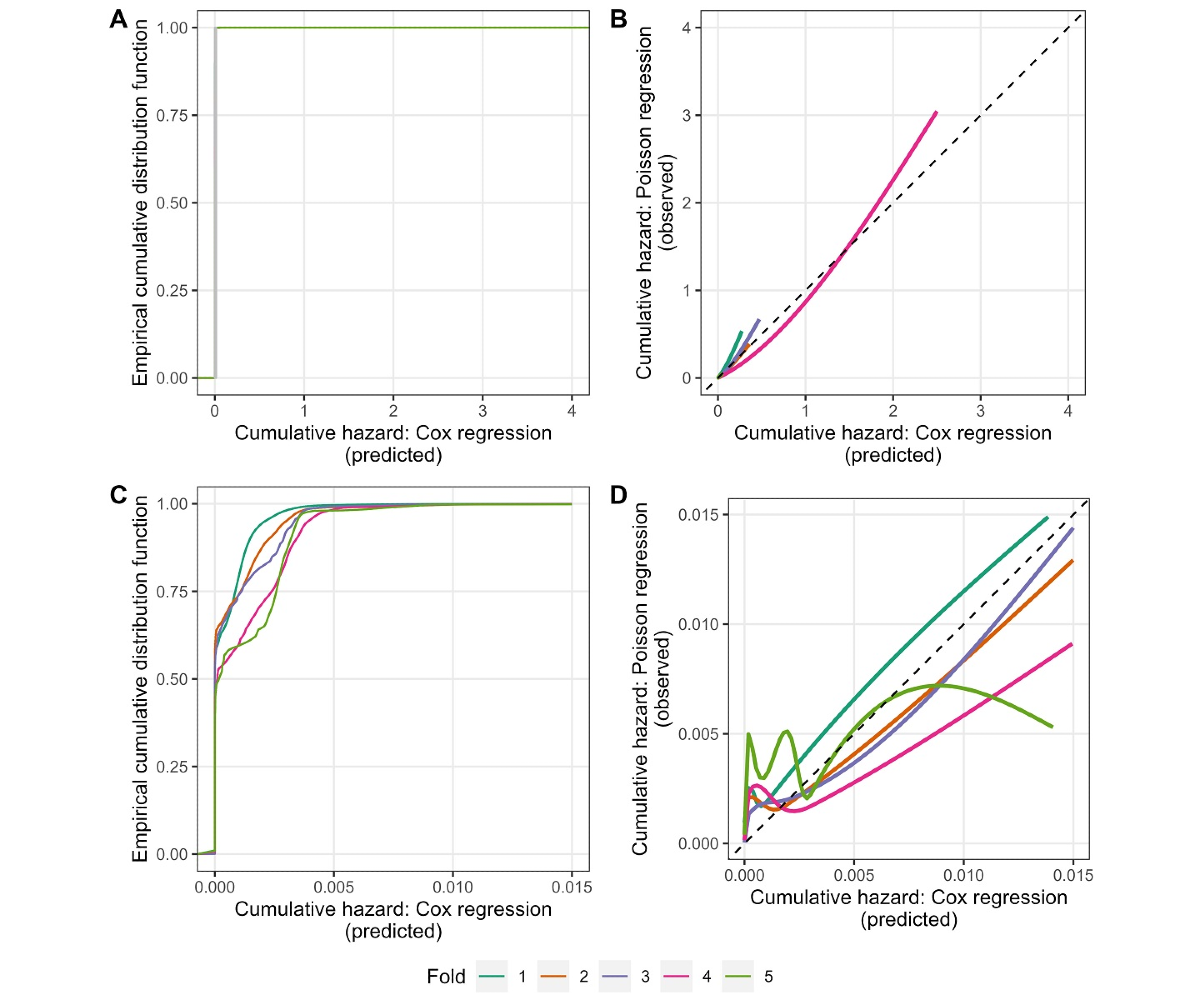
Calibration performance for each cross-validation fold. The top row (A and B) shows the full plots, and the bottom row (C and D) shows the same curves but with a focused view to better visualize the largest portion of predicted values (predicted cumulative hazards between 0 and 0.015). (A and C) Empirical cumulated density functions of the cumulative hazard from the Cox regression model. (B and D) Moderate calibration assessment as described by McLernon et al.

The AUC was 0.899 (95% CI 0.88-0.91) when a prediction time of 24 hours after admission was used. Discriminatory performance reduced as the admission progressed (Figure 1). There were substantial differences in performance between hospitals, particularly as time since admission increased. Model calibration plots indicated that overestimation and underestimation of risk occurred, with the degree of deviation from optimal calibration dependent on the cross-validation fold (Figure 2 [24]).

**Table 3:** Summary of the final model.

Parameter	Estimate (95% CI)	Hazard ratio (95% CI)
Female	–0.2189 (–0.2705 to –0.1673)	0.8034 (0.7630-0.8460)
Age (years)^a^	0.0221 (0.0153 to 0.0290)	1.0224 (1.0154-1.0294)
Age (years)^b^	–0.0051 (–0.0169 to 0.0068)	0.9949 (0.9832-1.0068)
Age (years)^c^	–0.0297 (–0.0802 to 0.0209)	0.9708 (0.9229-1.0211)
Time since 2018 (years)^a^	0.0228 (–0.0868 to 0.1324)	1.0231 (0.9169-1.1416)
Time since 2018 (years)^b^	–0.0370 (–0.3597 to 0.2857)	0.9637 (0.6979-1.3307)
Time since 2018 (years)^c^	0.0451 (–0.9285 to 1.0186)	1.0461 (0.3952-2.7692)
Admission source (community service)	0.0054 (–0.2627 to 0.2736)	1.0055 (0.7690-1.3147)
Admission source (emergency department - this hospital)	–0.0003 (–0.0828 to 0.0822)	0.9997 (0.9205-1.0857)
Admission source (other)	–0.5548 (–0.8137 to –0.2959)	0.5742 (0.4432-0.7439)
Admission source (outpatient department - this hospital)	–0.3924 (–0.5040 to –0.2807)	0.6755 (0.6041-0.7552)
Admission source (private medical practitioner [not psychiatry])	–0.7988 (–1.2337 to –0.3639)	0.4499 (0.2912-0.6950)
Admission source (residential aged care service)	–0.0465 (–0.3199 to 0.2270)	0.9546 (0.7262-1.2549)
Admission source (routine readmission no referral required)	–0.8621 (–1.1727 to –0.5516)	0.4223 (0.3095-0.5760)
Medical service (geriatrics)	0.2673 (0.1288 to 0.4059)	1.3065 (1.1375-1.5006)
Medical service (neurosurgery)	–0.0202 (–0.1929 to 0.1525)	0.9800 (0.8245-1.1648)
Medical service (orthopedics)	–0.3137 (–0.4298 to –0.1976)	0.7307 (0.6506-0.8207)
Medical service (other)	–0.5710 (–0.6393 to –0.5027)	0.5650 (0.5277-0.6049)
Medical service (palliative medicine)	0.2389 (0.0888 to 0.3889)	1.2698 (1.0928-1.4754)
Medical service (psychiatry)	–0.3366 (–0.4531 to –0.2201)	0.7142 (0.6356-0.8025)
Medical service (psychogeriatric)	–0.0213 (–0.3123 to 0.2697)	0.9789 (0.7317-1.3095)
Medical service (rehabilitation)	0.0417 (–0.0969 to 0.1802)	1.0425 (0.9076-1.1975)
Medical service (spinal)	–0.5627 (–1.0919 to –0.0335)	0.5697 (0.3356-0.9671)
Time since admission (hours)^a^	0.3008 (0.2454 to 0.3562)	1.3510 (1.2781-1.4279)
Time since admission (hours)^b^	–0.3621 (–0.5374 to –0.1867)	0.6962 (0.5842-0.8297)
Previous falls (n)	0.9053 (0.8640 to 0.9467)	2.4728 (2.3727-2.5771)

^a^Level 1 for the spline terms (refer to Multimedia Appendix 1 for knot locations for each term).

^b^Level 2 for the spline terms.

^c^Level 3 for the spline terms.

## Discussion

### Principal Findings

Using only routinely recorded information in EMR systems from 5 hospitals of varying capacities and capabilities, we successfully developed a novel prognostic model for inpatient falls using a time-to-event modeling approach. Our approach has the potential to add value to inpatient falls prevention efforts by updating prognostic risk predictions with new information throughout a patient’s admission as it becomes available without requiring clinical staff to collect additional fall-risk screening or assessment data and manually estimate risks. It was encouraging to observe useful predictive performance that was not inferior to more labor- and data-intensive fall risk prediction approaches that featured in our previous review [8]. Studies reporting AUC within our previous review that included external validation and used data from adult inpatients typically reported AUC in the range of 0.7 to 0.85 [30-32]. By developing our model using only data from EMR, this labor-efficient approach to estimation of inpatient fall risk has the potential to be implemented in clinical decision support systems that aid in appropriate allocation of effective inpatient fall prevention interventions without dependency on physical functional performance assessment [33,34].

Overall, our model demonstrates similar or greater discriminatory performance than many other published models for inpatient falls, particularly those that have been externally validated [2,8]. Calibration was site dependent but generally poor. The variable model performance, in terms of both discrimination and calibration, and its association with hospitals may be explained by differences in patient cohorts, fall rates, and the nature of care provided in each hospital.

The poor calibration performance may suggest that the absolute model predicted risk should be interpreted with caution. However, predictions are likely to remain useful for if the models were implemented in a manner that prioritized higher-risk patients (based on rank of estimated risk) toward a fall prevention intervention rather than applying a selected cutoff point above which the patient receives the intervention.

This approach would better take advantage of the high discriminatory performance without relying heavily on the need for the model to be well calibrated and for the selected cutoff point to be used on potentially biased estimates of risk. The use of a cutoff point may not work effectively due to the observed miscalibration based on local clinical contexts, but model recalibration by hospital could be beneficial [35].

We observed that there was a high level of between-hospital variability in ROC curves, particularly for prediction times at day 4 and later. We suspect that this is due to there being relatively few patients and fallers who are still admitted at that time. For example, the particularly unstable ROC curve for hospital 4 observed in Figure 1 had a median length of stay of 0.4 (IQR 0.2-2.1) days, with likely relatively few admitted patients, and very few admitted patients with falls, remaining after day 3. We suspect that the ROC curve for this hospital for these later prediction times is likely influenced by relatively few data.

While some aspects of our model are directly comparable to existing inpatient fall prediction models, several are unique. We have not identified any time-to-event prediction models in the literature that evaluate performance in terms of discrimination and calibration [8]. As binary prediction models typically identify fallers as patients who fell over the remaining time in the admission without time-varying covariates, perhaps the closest comparison from our study would be our model’s discriminatory performance at day 1 before time-varying covariates had influenced risk predictions. For this scenario, the discrimination of our model demonstrates superior performance during internal-external cross-validation. In addition, the advantages of a time-to-event approach include the ability to update predictions as the admission progresses and new data become available, and better incorporation of current length of stay into estimated risk.

We used internal-external cross-validation because this is the most appropriate way to evaluate a clinical prediction model when (1) data are available from multiple sites and (2) there are enough data at each site to ensure that models developed from each cross-validation fold are adequately powered [16]. Many clinical prediction model development studies may only rely on data from 1 to 3 sites. In our previous review, we found that only 2 of 51 studies used data from more than 3 hospitals to develop their model (43 of which only used data from a single hospital) [8]. When only a few sites, it may be impractical to conduct internal-external cross-validation as there would only be 2- or 3-folds, and potentially have too small a portion of the entire dataset for those models to be adequately powered. Fortunately, in our study, we had data from 5 sites and, of the 5 models fit during cross-validation, the lowest number of events per parameter was 135, which was much higher than the minimum number estimated by our sample size calculations (11.75).

For evaluation of the discrimination, we used the area under the time-dependent ROC curve. To create a single summary measure, we used vertical averaging of the 5-folds [23]. A potential limitation of this is that hospitals are equally influential on the “combined” curve and the final estimate of discrimination, there is no additional weight given to hospitals with more data than others. However, in this study, the models with the lowest performance were typically those evaluated on hospitals with fewer admitted patients and events, rather than the largest hospital (“Fold 1” curve in Figure 1), which was consistently the best performing.

Most existing approaches to predicting inpatient falls use data collected upon admission to estimate risk for the duration of the entire admission. This may lead to confusing interpretations; if clinicians interpret predicted risks as a function of admission time, this can be misleading. For example, a user may interpret the patient’s daily fall risk as the total estimated risk divided by the expected length of stay in days, which inappropriately assumes falls risk is evenly distributed over the entire admission. A patient may have a relatively high risk of falling on any given day, but if they are admitted for a condition that is associated with shorter lengths of stay, then a binary prediction model may predict a deceptively low risk. Time-to-event models avoid this potentially erroneous conclusion by accounting for length of stay inherently. We anticipate that a model of this level of discriminatory performance may be useful when allocating patients for fall prevention intervention, but this would need to be evaluated prospectively or possibly within a simulation study [36].

The use of machine learning methods for clinical prediction models has been increasing in popularity, including neural networks or tree-based ensemble models such as random forests or extreme gradient boosting [37,38]. There are variants of these approaches capable of handling time-to-event models but none, to our knowledge, have been applied to develop a prognostic model for falls. For example, random survival forests have been used to predict colorectal cancer prognosis [39], and “pycox” [40], an implementation of a Cox model for a neural network using *PyTorch*, has been used to predict in-hospital events including mortality and discharge [41]. There has also been a recent increase in popularity of sequence models, including attention models and recurrent neural networks, which can incorporate a sequence of input data of variable lengths and of irregular intervals [42]. We expect that combining these approaches with a Cox layer, as is used in “pycox,” may lead to the benefits of both being able to use time-varying covariates and being able to appropriately predict falls as a time-to-event outcome. A recent study has implemented a similar approach to fit a time-dependent Cox survival neural network to generate dynamic predictions, with example datasets including age-related eye disease and time-to-liver transplant [43]. The machine learning methods included in our recent review [8] were limited to those predicting falls as either occurring any time in the admission or within a fixed time horizon [44,45], but none used a time-to-event outcome. Consequently, it is difficult to know whether falls may be better estimated by the additional complexity and ability to estimate interaction effects within these methods.

We expect that by incorporating more time-varying predictors in a Cox model to predict falls may improve performance but may require a larger dataset with more falls to avoid overfitting. Another limitation of using this model is that it requires computation to estimate risk and cannot be easily implemented without an EMR, as other commonly used analog falls risk assessment tools can [33,46]. Although a reduction in data collection burden for health professionals is a strength of our model, it is a potential barrier to implementation in settings without the required levels of digital maturity.

**Conclusions**

In this study, we have presented a prognostic model using time-to-event modeling. We used internal-external cross-validation with data from 5 hospitals with notable heterogeneity to estimate its generalizable performance and present the final model that incorporated data from all 1,107,556 inpatient admissions. We anticipate that the reported model estimates may be adjusted to improve calibration performance at each hospital, and that this model has potential use for improving clinical care in the context of prompting the initiation of fall prevention strategies. However, evaluating our model in a simulated clinical environment or prospective clinical trial is a requisite step in determining whether the model is clinically useful. These studies may guide the implementation of the presented model within a decision support system for falls prevention strategies in hospital inpatient environments. Given the use of time-to-event modeling approaches, we also anticipate that machine learning methods that account for time-varying predictors may lead to improved discrimination and calibration.

## Appendix

Multimedia Appendix 1 Knot locations for restricted cubic spline terms in the final model.

Multimedia Appendix 2 Summary of model parameters, patients, and events used for each model fit during cross-validation.

## Abbreviations

AUC: area under the curve
EMR: electronic medical record
ROC: receiver operating characteristic
TRIPOD: Transparent Reporting of a Multivariable Prediction Model for Individual Prognosis or Diagnosis

## Data Availability

The data used for this study cannot be shared publicly due to ethical and privacy considerations; however, the data analysis scripts are available on a public GitHub repository[ 28].
