# Supplementary material for: An Electronic Medical Record–Based Prognostic Model for Inpatient Falls: Development and Internal-External Cross-Validation
Source: J Med Internet Res. 2024 Nov 13;26:e59634. doi: 10.2196/59634 (PMC11602763; doi:10.2196/59634)
Supplement: Multimedia Appendix 1 [file jmir_v26i1e59634_app1.docx]

**Multimedia Appendix 1.** Knot locations for restricted cubic spline terms in the final model.

| Term | Knot Number | Knot Location |
| --- | --- | --- |
| Age (years) | 1 | 14.00 |
|  | 2 | 49.00 |
|  | 3 | 71.00 |
|  | 4 | 90.00 |
| Time since 2018 (years) | 1 | 0.26 |
|  | 2 | 1.48 |
|  | 3 | 2.67 |
|  | 4 | 3.80 |
| Time since admission (hours) | 1 | 0.00 |
|  | 2 | 36.00 |
|  | 3 | 204.00 |
