# Supplementary material for: An Electronic Medical Record–Based Prognostic Model for Inpatient Falls: Development and Internal-External Cross-Validation
Source: J Med Internet Res. 2024 Nov 13;26:e59634. doi: 10.2196/59634 (PMC11602763; doi:10.2196/59634)
Supplement: Multimedia Appendix 2 [file jmir_v26i1e59634_app2.docx]

| Model^*^ | Inpatient admissions | Patient days (truncated at 14 days) | Falls (n) | Model parameters (n) | Events per parameter |
| --- | --- | --- | --- | --- | --- |
| Fold: 1 | 644,665 | 935,735 | 2,977 | 22 | 135 |
| Fold: 2 | 748,481 | 1,244,944 | 4,627 | 26 | 178 |
| Fold: 3 | 957,098 | 1,532,182 | 5,366 | 26 | 206 |
| Fold: 4 | 983,407 | 1,501,363 | 5,176 | 26 | 199 |
| Fold: 5 | 1,096,573 | 1,706,308 | 5,870 | 26 | 226 |
| Final | 1,107,556 | 1,730,133 | 6,004 | 26 | 231 |
| ^*^Model represents the cross-validation fold models and the final model fit with all patient data. The fold models are those fit during internal-external cross-validation and incorporate all patient data except for the associated hospital of the same number. For example, the 'Fold: 1' model was fit using patient data from hospitals 2 to 5, with hospital 1 being the validation set. | | | | | |
